# Right Ventricular Myxoma Involving the Chordae Tendineae: A Case Report and Literature Review

**DOI:** 10.7759/cureus.74487

**Published:** 2024-11-26

**Authors:** Rishab Makam, Ayush Balaji, Akshay Balaji, Ramanish Ravishankar, Natasha Bocchetta, Mohamed Sherif, Mahmoud Loubani

**Affiliations:** 1 Department of Cardiothoracic Surgery, Castle Hill Hospital, Cottingham, GBR; 2 Department of Medicine, Hull York Medical School, Hull, GBR; 3 Department of Cardiothoracic Surgery, Hull York Medical School, Hull, GBR; 4 Department of Public Health, London School of Hygiene and Tropical Medicine, London, GBR

**Keywords:** cardiac tumours, decision making process, imaging, mediastinal cyst, myxoma

## Abstract

This case report describes a rare instance of massive right ventricular myxoma (RVM). A 36-year-old woman initially presented with progressive breathlessness and chest heaviness. Imaging revealed a large mass in the mediastinum, which was initially thought to be a pericardial cyst, and it was unclear whether the mass was intracardiac or extracardiac. Extensive investigations and multiple discussions concluded that the mass was a right ventricular mass. Intraoperatively, the mass was confirmed to be in the right ventricle (RV), with involvement of the chordae tendineae and an extremely thinned-out right ventricular free wall. Surgical excision of the tumour from the RV, tricuspid valve repair, and plication of the free wall were performed, leading to significant symptomatic relief. The mass was identified as a myxoma through histological diagnosis. This case highlights the diagnostic challenges and management strategies for RVM.

## Introduction

Cardiac myxomas (CMs) are the most common type of primary cardiac tumours in adults, accounting for more than 50% of cases. Typically arising in the atria, about 75% occur in the left atrium (LA) and 15%-20% in the right atrium (RA). Ventricular myxomas are extremely rare, comprising less than 5% of all CMs [1,2]. However, some evidence suggests that this proportion is higher in children, with one study reporting up to 20% [3]. These tumours typically arise from mesenchymal stem cells in the fossa ovalis or adjacent endocardium, linked embryologically to endocardial and epicardial cells. Two main histological types exist: polypoid myxomas, which are solid, smooth, and often associated with embolic events, and papillary myxomas, which are softer with friable projections, leading to higher embolisation risks. Sporadic forms differ from familial types, like Carney complex-associated tumours, which are multicentric and prone to recurrence [4]. Cardiac tumours pose significant diagnostic and therapeutic challenges due to their rarity and potential for severe complications, such as embolism, obstruction, and heart failure.

This report presents a case of right ventricular myxoma (RVM) in a young woman, discussing the clinical presentation, diagnostic approach, surgical management, and postoperative outcomes.

## Case presentation

**Figure 1:**
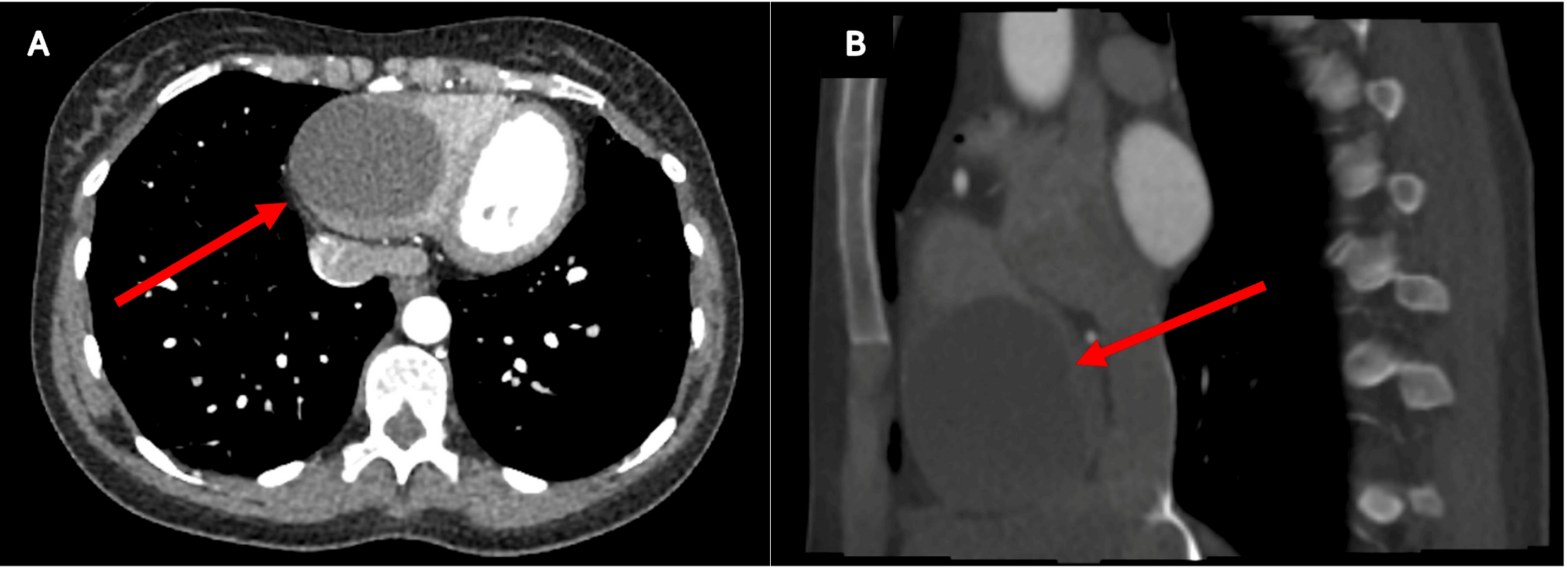
Preoperative CT scan showing a right ventricular mass. Images (A) and (B) show the transverse and sagittal views of the mass, respectively. CT, Computed tomography

A 36-year-old woman presented with acutely progressive breathlessness and constant chest heaviness. A computed tomography pulmonary angiography (CTPA) revealed a pulmonary embolism (PE) and a 6.2 cm cystic lesion near the right cardiophrenic angle, initially thought to be a pericardial cyst (Figure 1). Following the Multidisciplinary Team (MDT) discussion, the impression of external compression of the right ventricle (RV) led to the initial hypothesis of a pericardial mass. However, given the diagnostic uncertainty, the MDT recommended further imaging.

**Figure 2:**
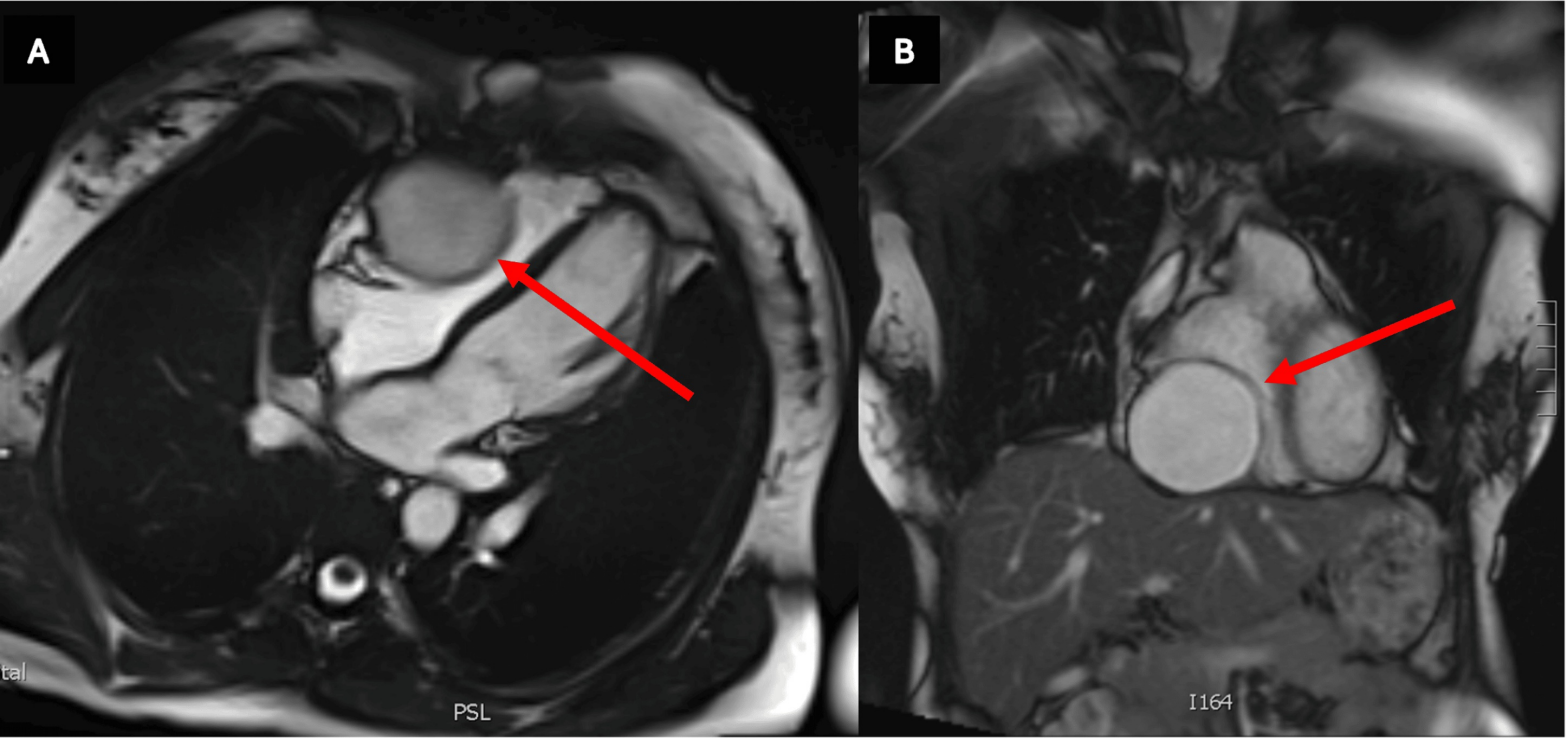
Preoperative MRI of myxoma. Images (A) and (B) show the transverse and coronal views of the mass, respectively. MRI, Magnetic resonance imaging

The echocardiogram revealed an increase in cyst size, now measuring 6.8 x 6.2 cm, causing compression of the basal to mid RV and RA. The echocardiographic study further showed the close relation and compression of the RV by the mass (Video 1). Subsequent magnetic resonance imaging (MRI) suggested that the lesion was more likely located within the RV rather than in the pericardial space, albeit inconclusive due to its proximity to the ventricular wall (Figure 2). The imaging studies also presented a unique diagnostic dilemma due to the cyst-like appearance and lack of a peduncle or cardiac attachment. Differential diagnoses included a myxoma, bronchogenic cyst, lipoma, or a primary malignant tumour.

**Video 1:** Preoperative TTE of the mass showcasing compression and distortion of the right ventricle. TTE, Transthoracic echocardiogram

The MDT concluded that surgical excision of the mass was necessary due to progressive symptoms, increased size, and clinical complications. This decision was made to alleviate the patient's symptoms and obtain a definitive histopathological diagnosis.

Following median sternotomy, cardiopulmonary bypass (CPB) was established using bicaval cannulation. An incision was made in the RA, and the RV was accessed through the tricuspid valve (TV). The mass was found to be invading the RV wall and extending to the papillary muscle, but sparing the ventricular septum. The invasion into the ventricular free wall was so significant that the tumour was visible through a translucent layer of the myocardium. The tumour was carefully excised, and the ventricular wall was plicated using Teflon strips. The tumour, extending to the septal and posterior papillary muscles, was carefully excised, including the chordae tendineae. The RV was then washed out, and the papillary muscles were reconnected to the leaflets using Gore-Tex Neochords (W. L. Gore & Associates, Newark, DE, USA). A 28 mm Edwards Physio tricuspid ring (Edwards Lifesciences, Irvine, CA, USA) was inserted with Ethibond sutures (Ethicon, Inc., New Brunswick, NJ, USA). Intraoperative transoesophageal echocardiogram (TOE) showed only trivial to mild tricuspid regurgitation. Finally, the RA was closed after ensuring no residual tumour in the nearby chambers.

**Figure 3:**
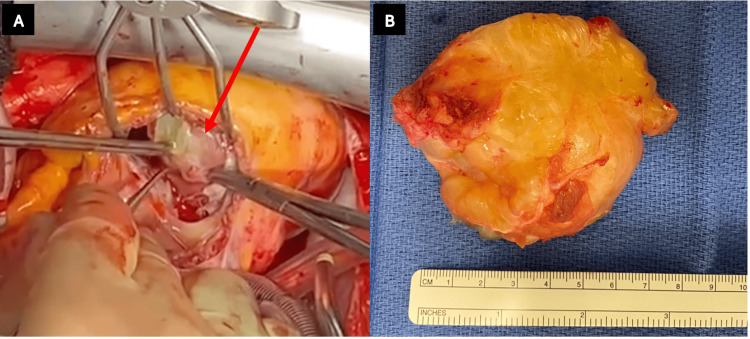
Pane (A) shows an intraoperative image of the mass within the right ventricle, and Pane (B) shows the resected mass measurements post-excision.

The diagnosis of RVM was confirmed through histopathological examination. The mass measured 70 x 60 x 40 mm (Figure 3). The postoperative period was uneventful, and the patient showed significant improvement. Follow-up imaging and clinical evaluations over the next year revealed no recurrence.

## Discussion

CMs may be the most common intracardiac tumours, but they can present with a variety of symptoms, depending on their location and potential for embolisation. Constitutional symptoms include systemic features, such as fever and weight loss. Studies have noted that these symptoms are a product of interleukin-6 production by the myxoma and resolve following resection [4-7]. Embolic events, such as stroke and PE, are related to the location of the tumour, with left-sided lesions leading to systemic emboli and right-sided lesions tending to cause pulmonary emboli. Finally, cardiac or obstructive symptoms are a function of the myxoma’s mass effect, obstructing the atrial or ventricular inlets and outflow tracts [1,2,8]. For patients with left-sided myxomas, the obstruction may lead to pulmonary congestion or systemic ischaemia, causing dyspnoea or syncope. Patients with right-sided myxomas, instead, present with peripheral oedema, ascites, or superior vena cava syndrome [9].

RVMs often present differently from their more frequent atrial counterparts. These tumours can obstruct the right ventricular outflow tract, leading to symptoms of exertional dyspnoea, fatigue, and syncope. Embolic phenomena are less common compared to left atrial myxomas but can occur, as exemplified by this case. Studies have also reported ventricular myxomas attached to the cardiac anatomy, causing valvular regurgitation [10,11].

Transthoracic echocardiography (TTE) is typically the initial diagnostic tool, providing valuable information about the size, location, and tumour mobility. TOE offers more detailed visualisation, especially for smaller or more complex lesions. Combined, they have an accuracy rate of up to 96% when diagnosing myxomas [2]. Cardiac MRI and CT scans are used for precise anatomical assessment, surgical planning, and to rule out differential diagnoses. Definitive diagnosis, however, requires histopathological examination. Myxomas are usually composed of stellate or fusiform cells layered within a myxoid extracellular matrix of peptidoglycans, elastin, and collagen. Typically, myxomas exhibit positivity for endothelial markers such as CD31, CD34, and FVIIIAg [4,12].

RVMs pose challenges due to their rarity and atypical presentation. They carry a significant risk of morbidity and mortality when left untreated. Surgical resection remains the primary treatment, and advancements in surgical techniques and postoperative care have improved outcomes. With a low surgical mortality rate and recurrence of less than 5%, prompt intervention is crucial to prevent severe complications. A comprehensive assessment of the invasion site, using better imaging and complete resection, is crucial for a successful outcome. The use of a bronchoscope is commonly employed to improve the ventricular apex view, but a recent report illustrated the utilisation of intraoperative choledochoscopy in examining the ventricular wall [13]. Shah et al. describe the importance of using an endocardial button to maximise resection and reduce the recurrence rate [14,15].

## Conclusions

Current literature relies heavily on case reports and small series, due to the rarity of RVMs. Future research should focus on developing standardised guidelines for managing these rare tumours. The detailed preoperative investigation strategy and operative procedure highlight the importance of careful surgical planning, to achieve optimal outcomes in such complex cases.
